# Optimized imaging methods for species-level identification of food-contaminating beetles

**DOI:** 10.1038/s41598-021-86643-y

**Published:** 2021-04-12

**Authors:** Tanmay Bera, Leihong Wu, Hongjian Ding, Howard Semey, Amy Barnes, Zhichao Liu, Himansu Vyas, Weida Tong, Joshua Xu

**Affiliations:** 1 Division of Bioinformatics and Biostatistics, National Center for Toxicological Research (NCTR), Food and Drug Administration (FDA), Jefferson, AR 72079 USA; 2 Food Chemistry Laboratory-1, Arkansas Laboratory (ARKL), Office of Regulatory Sciences, Office of Regulatory Affairs (ORS/ORA), FDA, Jefferson, AR 72079 USA

**Keywords:** Biological techniques, Ecology

## Abstract

Identifying the exact species of pantry beetle responsible for food contamination, is imperative in assessing the risks associated with contamination scenarios. Each beetle species is known to have unique patterns on their hardened forewings (known as *elytra*) through which they can be identified. Currently, this is done through manual microanalysis of the insect or their fragments in contaminated food samples. We envision that the use of automated pattern analysis would expedite and scale up the identification process. However, such automation would require images to be captured in a consistent manner, thereby enabling the creation of large repositories of high-quality images. Presently, there is no standard imaging technique for capturing images of beetle elytra, which consequently means, there is no standard method of beetle species identification through elytral pattern analysis. This deficiency inspired us to optimize and standardize imaging methods, especially for food-contaminating beetles. For this endeavor, we chose multiple *species* of beetles belonging to different *families* or *genera* that have near-identical elytral patterns, and thus are difficult to identify correctly at the *species* level. Our optimized imaging method provides enhanced images such that the elytral patterns between individual species could easily be distinguished from each other, through visual observation*.* We believe such standardization is critical in developing automated species identification of pantry beetles and/or other insects. This eventually may lead to improved taxonomical classification, allowing for better management of food contamination and ecological conservation.

## Introduction

Due to associated health risks, food products are regularly inspected for pantry beetle infestation^1–4^. The health-risk level involved in such contamination depends on the species of beetle, as some species are more harmful than others^5–7^. Inability to distinguish between pantry pests, agricultural pests and pests that are pathogenic vectors could adversely affect regulatory decisions, to grave consequences. Therefore, accurate and expeditious species-level identification of food-contaminating beetles is essential in assessing the actual risk, and thus for better managing contamination^8^. The current method for identifying the *genus* or *species* (whenever possible) of food contaminating beetles is done manually by expert entomologists who rely mostly on visual observation of anatomical details of the insect or its fragments^9,10^. This makes the process highly dependent on the expertise of the analyst, prone to personal bias and therefore less scalable.

As an alternative, researchers have proposed chemical methods for species identification, which show great promise^11–13^. However, regulatory bodies depend on methods of visual inspection and subsequent analysis, most likely due to the decreased possibility of false positives or negatives^14^. Thus, we propose that species identification could be done by characterizing elytral patterns which, like human fingerprints, are unique to each species^15^. As one of the hardest insect body parts, elytra may break into fragment, but do not become completely pulverized during food processing and/or sample preparation steps^16^. The use of patterns, not anatomical cues (such as shape and size of the body parts such as elytra, antenna or legs), also opens up the possibility of automating the whole process by using computational image analysis algorithms. This process might significantly reduce the time and manpower required for species identification, as observed in similar studies from other fields^17–20^.

In previous works, we demonstrated that food-contaminating beetles could be identified by automated pattern recognition using analyzed elytra images from 15 different species of pantry beetles^21–24^. In these studies, we noticed that some beetle species consistently were not being predicted accurately and were difficult to identify correctly, they were misidentified as other species, despite the use of state-of-the art pattern recognition algorithms. For instance, *Lasioderma serricorne* (*L. serricorne*) and *Stegobium paniceum* (*S. paniceum*)*,* both belonging to the family *Anobiidae,* consistently were predicted inaccurately and often were confused with each other. The same trend was observed between the species *Tribolium castaneum* (*T. castaneum*) and *Tribolium confusum* (*T. confusum*) of *genus Tribolium* (*Family*: *Tenebrionidae*) and *Oryzaephilus mercator* (*O. mercator)* and *Oryzaephilus surinamensis (O. surinamensis)* of *genus Oryzaephilus* (*Family: Silvanidae*). Upon further investigation, we realized that the images used for their identification contained such artifacts as glare spots, which were interpreted incorrectly as actual features in elytral patterns. The image set simply lacked the quality and clarity to reveal finer details of interest, and therefore, failed to yield species level accuracy, especially for species with near-identical elytral patterns. This is consistent with similar studies on species identification or pattern recognition and highlights the necessity of a high-quality image set for achieving good prediction accuracies^25–28^. This compelled us to develop a better imaging method which could yield elytral images of sufficient detail to enable us to distinguish between species from the same *family* or *genus*.

In this work, we focused on optimizing imaging conditions in order to capture elytral images with great clarity and quality. We used seven different beetle species such as *L. serricorne* and *S. paniceum* of the family *Anobiidae; Gnatocerus cornutus* (*G. cornutus*) and *T. castaneum* of the family *Tenebrionidae;* and *T. castaneum* & *T. confusum* of genus *Tribolium* (both belonging to the family *Tenebrionidae*) and *O. mercator* and *O. surinamensis* of genus *Oryzaephilus.* Three different lighting systems, namely, two-point reflected light (*2Pt_Rf*), reflected ring light with polarizing filter (*RRf_P*) and transmitted light (*Trans*), were utilized as illumination sources to compare their performances in distinguishing species of the same *family* or *genus*. The most conventional lighting system, *2Pt_Rf* , was used in our previous studies, but showed significant reflections and glare spots from the waxy coating that naturally exists on elytral surfaces. Images obtained using the *RRf_P* system did not obstruct elytral patterns and helped us understand the effect of glare in images.

The *Trans* system revealed the internal structure of elytra, and compared to the reflected light, showed surface features more clearly. Imaging magnification varied between 20 × to 160 × to balance between the field of view and pattern size. To include all the patterns, both dorsal (D) and ventral (V) sides of elytra were imaged for different illuminations and magnifications and matched to the optimum imaging conditions. This method therefore was aimed at producing high-quality images in an efficient and consistent manner, which often is the prerequisite for automated species identification through image analysis using machine learning. In the future, we anticipate this will help in building a database of images for pantry beetles (or other orders of insects) that would facilitate automated species identification using elytral pattern recognition and may eventually help better manage contamination scenarios and ecological systems in real-world situations.

## Experimental methods

### Elytra harvest

Samples of pantry beetles of all seven species, which previously had been identified by expert entomologists, were cataloged and preserved in ~ 80% ethanol (in deionized water). They then were dissected under a microscope (Leica EZ3) to harvest their elytra. The elytra were cleaned in ethanol and deionized water through mild sonication, and subsequently air-dried before being moved under the microscope for imaging. No structural degradation, including the dissolution of the waxy coating on the elytra, was observed during these sample preservation and preparation steps. The list of beetle species and their scientific names, common names and abbreviations used here are listed in Table 1.

**Table 1:** The various species imaged in this study along with their *genus*, *family* and common names and the abbreviations used in this study.

No	Family	Genus	Species	Common name	Abbrev
1	*Anobiidae*	*Laisoderma*	*serricorne*	Cigarette Beetle	*LS*
2		*Stegobium*	*paniceum*	Drugstore Beetle	*SP*
3	*Tenebrionidae*	*Gnatocerus*	*cornutus*	Broadhorned Flour Beetle	*GC*
4		*Tribolium*	*castaneum*	Red Flour Beetle	*TCa*
5		*Tribolium*	*confusum*	Confused Flour Beetle	*TCo*
6	*Silvanidae*	*Oryzaephilus*	*mercator*	Merchant Grain Beetle	*OM*
7		*Oryzaephilus*	*surinamensis*	Sawtoothed Grain Beetle	*OS*
8	*Chrysomelidae*	*Zabrotes*	*subfasciatus*	Mexican Bean Weevil	
9	*Bostrichidae*	*Rhyzopthera*	*dominica*	Lesser Grain Borer	
10	*Curculionidae*	*Sitophilus*	*granarius*	Granary Weevil	
11		*Sitophilus*	*oryzae*	Rice Weevil	
12	*Tenebrionida*	*Tribolium*	*freemani*	Kashmir Flour Beetle	
13		*Tribolium*	*madens*	Black Flour Beetle	

### Imaging

**Figure 1:**
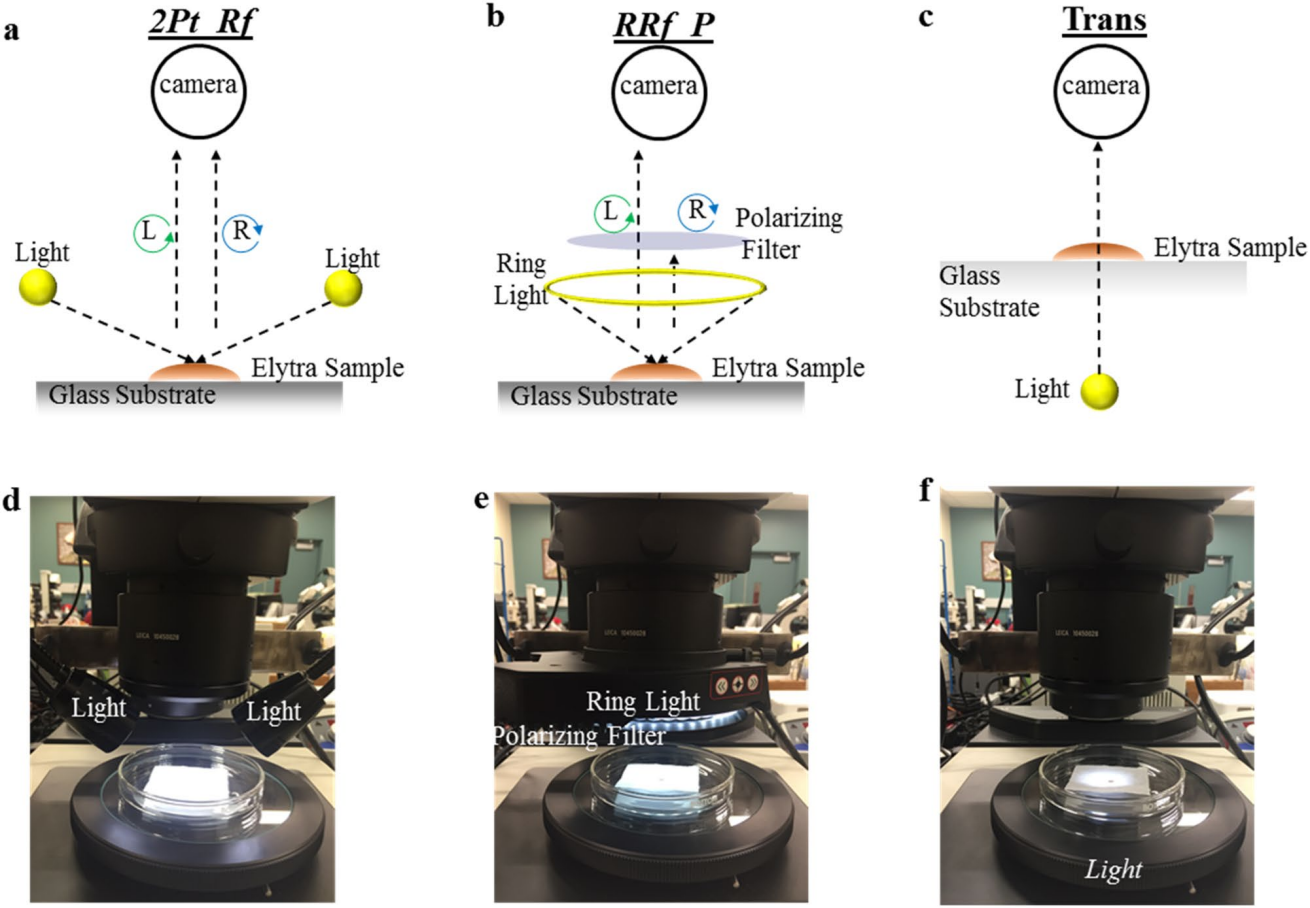
Schematic and experimental setup for three lighting conditions used in this study; **(a–c)** schematic ray-diagram for the imaging conditions and **(d–f)** actual laboratory setup.

Given that elytra are curved objects, we used a confocal microscope (Leica MA 205) for their imaging. Images were captured under three different lighting conditions (all bright field imaging using white light without any fluorescent filters) namely, *2Pt_Rf*, *RRf_P* and *Trans*. For the *2Pt_Rf* system (LED Stereo Microscope Light Source), two LED based point light sources were used to illuminate the samples from their left and right sides, as is done in most filth laboratories^9^. For the *RRf_P* setup, we used a ring light (Leica LED5000RL) which provided more uniform illumination to the samples. The ring light was attached to a polarizing filter (Leica PF) which, when adjusted, removed most of the glare from the images. The *Trans* setup simply used a LED light source located at the base of the microscope on which the samples were placed. For any of these conditions, choosing the correct light intensity is important for avoiding any over or under exposure. All three conditions are illustrated, both schematically and with the actual experimental set-up, in Fig. 1.

To image curved elytra, we performed Z-stack imaging. In this case, multiple images were captured at various focal planes between the top and bottom most planes of the elytra. Selecting the top and bottom image planes accurately was critical. Fixing the planes too far away from the actual planes produced more image distortions and fixing them smaller than the actual range produced blurriness and loss of features. It is advisable to set the interplanar distances between two images to ~ 10 µm to ensure that all intricate pattern details are included during imaging. Multiple raw images obtained from various focal planes then were processed using the Leica suite’s built-in Z processing algorithms that yielded a 2D equivalent image of the 3D elytra. To capture the images, a Leica MC170HD camera was used with an image resolution set to 2592 × 1944 dpi (the highest resolution available). In this study, both dorsal and ventral sides of each elytron were imaged at three lighting conditions and at magnifications varying from 20x to 160x. At the end of the imaging exercise we collected 20 images that collectively represented each species under different imaging condition (three lighting at four different magnification ranging from 20x to 160x, each for all seven species).

### Image analysis using ImageJ

Images captured using all three lighting conditions were analyzed through ImageJ to grossly estimate the area of glares and number of glare spots. This was done simply through steps such as ‘threshold’, ‘binary’ and ‘analyze particles’ that yielded the total area and number of points due to over-exposure. Average values were obtained by analyzing 10 elytra per *species* and plotted for comparison. For FFT (Fast Fourier Transformation), first a portion of the images that shows elytral patterns clearly were selected (to avoid any background signal). They were then converted to their FFT images using the FFT function (under Process Tab). For the pattern analysis, images captured only at 100x in *Trans* set up (being the optimal set up) were used. There were analyzed to calculate the ‘size’ and ‘shape’ distribution of the elytra patterns. First, the central portion of the raw images that showed the patterns only (without background or edges) were cropped manually to create the input image folder. They were processed for ‘size’ and ‘circularity’ calculation using ImageJ, in a manner similar to our earlier reports^29^. The sequential steps used for the analysis are ‘set scale’, ‘threshold’, conversion to ‘binary’, ‘despeckle’ and ‘analyze particles’ and have been illustrated schematically in the supplementary information (Supplementary Figure S2). The ‘circularity’ and ‘size’ distributions were binned in histograms and then analyzed through log-normal distribution to obtain parameters such as mean and standard deviations (SD).

## Results and discussion

**Figure 2:**
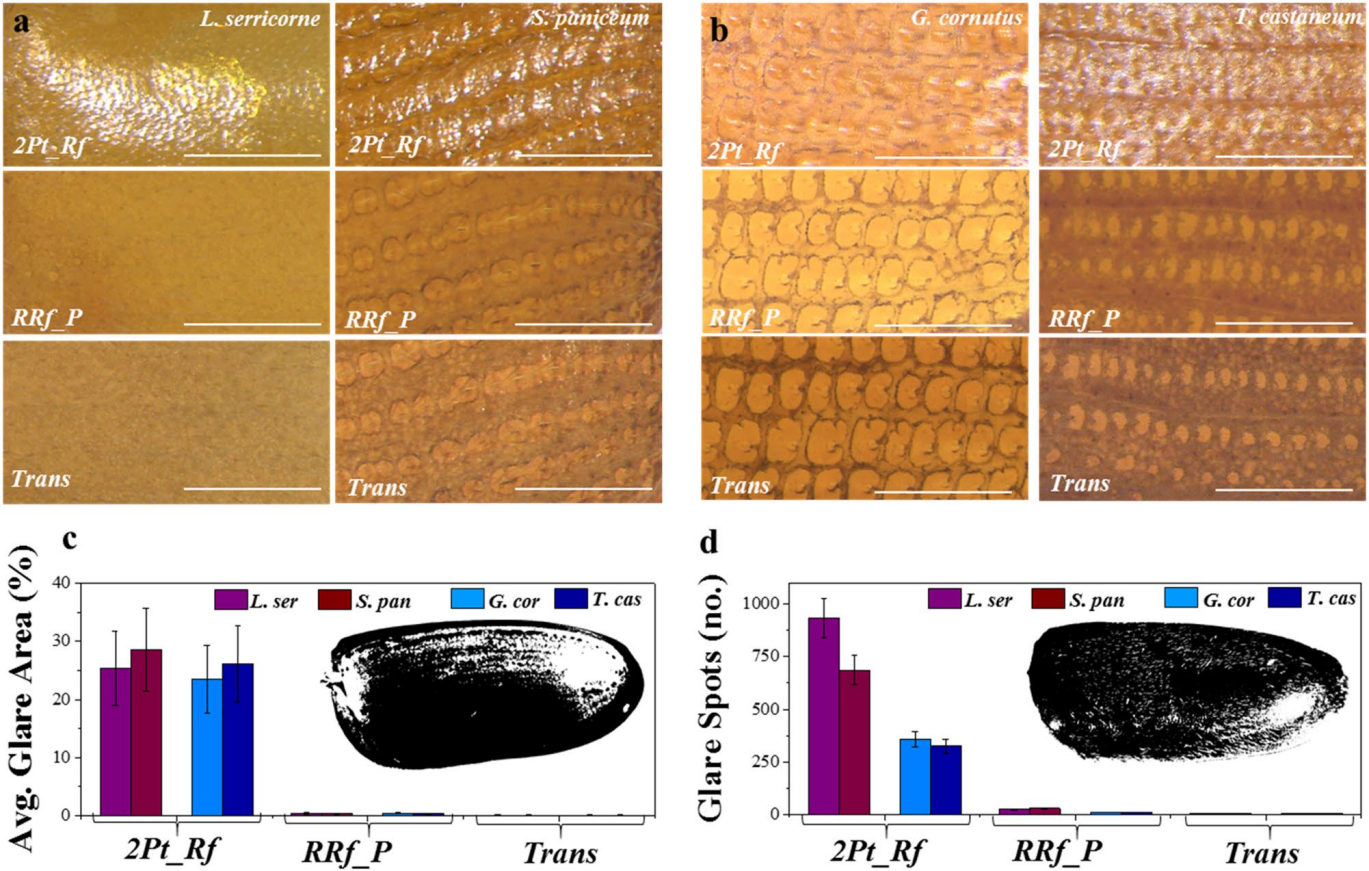
Comparing three different illumination systems. Images of elytra under three different lighting conditions (top: *2Pt_Rf*, middle: *RRf_P* and bottom: *Trans*), for two different families **(a)** *Anobiidae,* with species *L. serricorne* (1st column) and *S. paniceum* (2nd column) and (**b)** *Tenebrionidae* with species *G. Cornutus* (3rd column) and *T. castaneum* (4th column). All images were captured at 50x magnification, with the scale bars being 250 µm. The glare in images due to *2Pt_Rf* illumination is evident. The *RRf_P* and *Trans* systems provide a much better alternative that significantly reduces glare, which obstruct elytral patterns. The extent of glare has been quantified as **(c)** percentage of elytra area obstructed due to glare, **(d)** number of glare spots (i.e. artifacts) due to setae when compared against three different illumination systems for two different *families,* which further highlights the advantages of *RRf_P* and *Trans* systems.

The illumination system often is considered the key setting in most image acquisitions^30^. Thus, we first studied this system under three different settings, namely *2Pt_Rf*, *RRf_P* and *Trans* due to their practical and scientific merits. The schematic ray diagrams showing the lighting conditions and their experimental set-ups have already been illustrated (Fig. 1). The *2Pt_Rf* system is one of the most-used illumination systems in the field of entomology and food-filth detection, for it allows the operator to view a wide area of samples, even when they are dark and thick^9^. Beetle elytra (and other external surfaces of beetles) are coated with natural waxy substances and sometimes also with setae (hair-like features), which scatter reflected light, causing glare spots in their images^31^. Due to its reflective nature, this system, produced significant glare spots, often overshadowing the actual patterns of the elytra as seen on the top rows of Fig. 2a,b, and rendering it the least advantageous for our application.

The use of *2Pt_Rf* light produced so much glare that it obstructed about 25% of the pattern area (Fig. 2c). Moreover, the *dorsal setae* produced a large number of tiny glare spots that could easily be confused for feature points (Fig. 2d), and led to misidentifications in previous studies^21–24^. Furthermore, we observed that the position of the glare spots did not remain constant, and varied with the angle of incident light and/or the ambient lighting conditions (Supplementary Figure S1). Such randomness in image illumination (*i.e.*, brightness and contrast) makes this setting very inconsistent, (*e.g.,* highly dependent on random, external factors) and rendering it ineffective for automated pattern analysis, particularly in developing computer models for pattern recognition^27,28^.

The *RRf_P* system was a significant improvement over the *2Pt_Rf* system, as it allowed us to remove many of the illumination inconsistencies (Fig. 2a,b, the middle row). The use of a ring light instead of two light bulbs provided a more uniform illumination, and the polarized filter helped minimize glare spots from the images by imaging in circular polarized light (also minimizing the incoherent scattering of light, responsible for glare)^32^. However, upon closer observation, we found that the illumination system could not completely remove all the glare spots, especially for the species with dorsal setae. The orientation of the polarizing filter can be adjusted to remove random scattering of light only from one particular plane and/or angle. Since *dorsal setae* lie at a slightly different angle to the elytra surface, it was not possible to simultaneously remove glare spots originating from both elytra and dorsal setae. However, this setting was found to be excellent in imaging whole insects and/or other thick, dark or shiny objects that may have been challenging when using the *2Pt_Rf* setting^33^.

Compared to *RRf_P* and *2Pt_Rf*, the *Trans* system was a much simpler and more economical set-up that produced excellent glare-free images (Fig. 2a,b, the bottom row). The images of elytra revealed patterns clearly as they did not suffer from any reflective glares (Fig. 2c,d). Unlike the *RRf_P* system, images captured in *Trans* remained mostly unaffected by the polarizing filter or its orientation (Supplementary Figure S2). This makes *Trans* least susceptible to variations such as those in operating personnel and ambient lighting conditions; and it remained relatively unaffected by the change in intensity of the transmitted beam, (i.e., due to the camera’s automatic intensity balance setting). Methods that enable consistent acquisition of data are highly desirable for any automated processing as they help avoid any batch effect or unknown error^34,35^. In this regard, the *Trans* system provides an effective, yet simple solution for consistently capturing clear, high-quality images.

We also captured images under the combined illumination of *RRf_P* and *Trans* systems. This combined lighting system, however, fell short by revealing both surface and internal features with equal precision, and its images more closely resembled the *RRf_P*-based images (Supplementary Figure S3). It was not possible to balance or equalize the illumination intensities of the two (i.e., reflected and transmitted) light beams. The ring light was higher in illumination intensity compared to the transmitted beam, which also underwent sample adsorption, possibly resulting from the camera’s greater expose to reflected light, producing images similar to those captured using *RRf_P*.

The key to discerning between similar patterns lies in acquiring images that enable the visualization of fine pattern details. This would permit maximum information to be extracted from each image and could help identify a species with a much larger set of characteristic features. To ensure visualization of the most patterns, both sides (D and V) of elytra were imaged. We noted that both of these illumination systems were excellent in revealing the pattern details, as one can clearly observe differences in patterns for D and V sides of the same elytron. The lack of glare spots made distinguishing between beetles belonging to the same *family* relatively easy, as their pattern features such as color, design, and setae are quite different from each other when observed through either the *RRf_P* or *Trans* illumination systems (Supplementary Figure S4).

**Figure 3:**
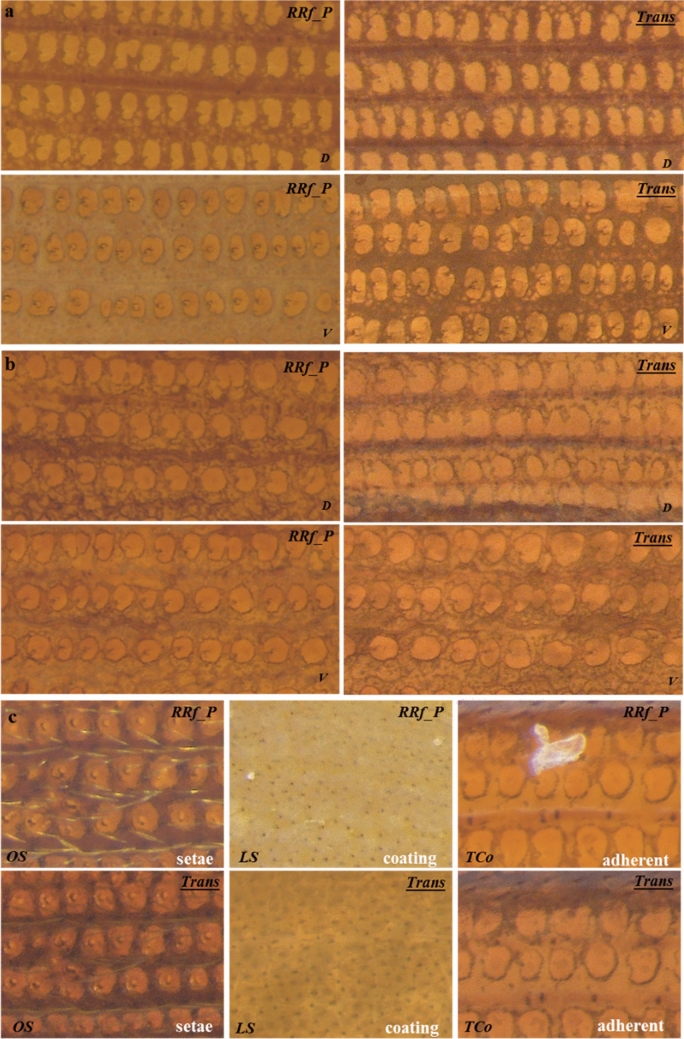
Acquired patterns on either side of elytra dorsal (D) & ventral (V) sides for maximum visualization of the patterns and to compare the two different illumination systems, *RRf_P* and *Trans*, for **(a)** *T. castenium*, **(b)** *T. confusum* both belonging to the *genus Tribolium*; and are well known for their remarkably similar appearance; and **(c)** in visualizing surface features in *O. surinamensis* (OS), *L. serricorne* (LS), and *T. confusum* (TCo). Although, both systems seemed equally good for visualizing elytral patterns, the *Trans* system was found to be the least affected by minor surface features such as setae, thin surface coatings or occasional adherents, making it more advantageous for the imaging application.

Differences between species were subtler for beetles belonging to the same *genus* however. For instance, rectangular patterns are only slightly lighter with marginally thicker ridge lines between *T. castaneum* and *T. confusum* (both *genus Tribolium*), irrespective of the illumination system used (Fig. 3a,b). Differences also were found to be subtle between *O. mercator* and *O. surinamensis* (*genus Oryzaephilus*) (Supplementary Figure S4e). For the same species, the variation in elytra patterns due to two different illumination settings (*RRf_P* and *Trans*) could only be perceived during careful observation of the images (refer to first row for D & second row for V side in images Fig. 3a,b). This difference, though minute, originated from the variation in the imaging mechanism and deserves comprehensive interpretation. The *RRf_P* system uses a light beam that reflects back from the specimen surface to capture an image, which allows better visualization of surface features. On the contrary, the *Trans* system uses a transmitted light beam that traverses through the specimen allowing better visualization of internal structures, in our case this is the cytoskeletal pattern on each elytron. That may explain why the images captured through the *Trans* system did not appear significantly different between the D and V sides, as they did for *RRf_P* lighting (Fig. 3a,b).

In an ideal scenario the *RRf_P* system should be used for imaging surface features such as setae, contours and the like, and the *Trans* system for such internal structures as ridges and bulges. In practice however we observed that the *RRf_P* system is more susceptible to dorsal setae or artifacts such as contaminants or adherents (Fig. 3c). In several of the elytron samples, especially in the case of deeply concave samples such as *L. serricorne*, we found thin adherent coatings (possibly from the medium in which the insect lived) on the V side of the elytra persisted even after thorough ultrasonic cleaning. We also observed occasional dust-like adherent on either side of the elytra, all of which masked the actual elytral patterns. Additionally, we foresee the loss of non-structural surface features such as *setae* in real-world samples that undergo aggressive sample preparation steps prior to analysis. The internal structures of elytra are made of well crosslinked chitin (or its modified version), which is known to have excellent mechanical and structural properties^16,36^. Thus, capturing images through the *Trans* system is advantageous in this regard, as it captures the internal structures which usually remain unaffected by minor surface adhesions, contamination or even vigorous sample preparation steps.

**Figure 4:**
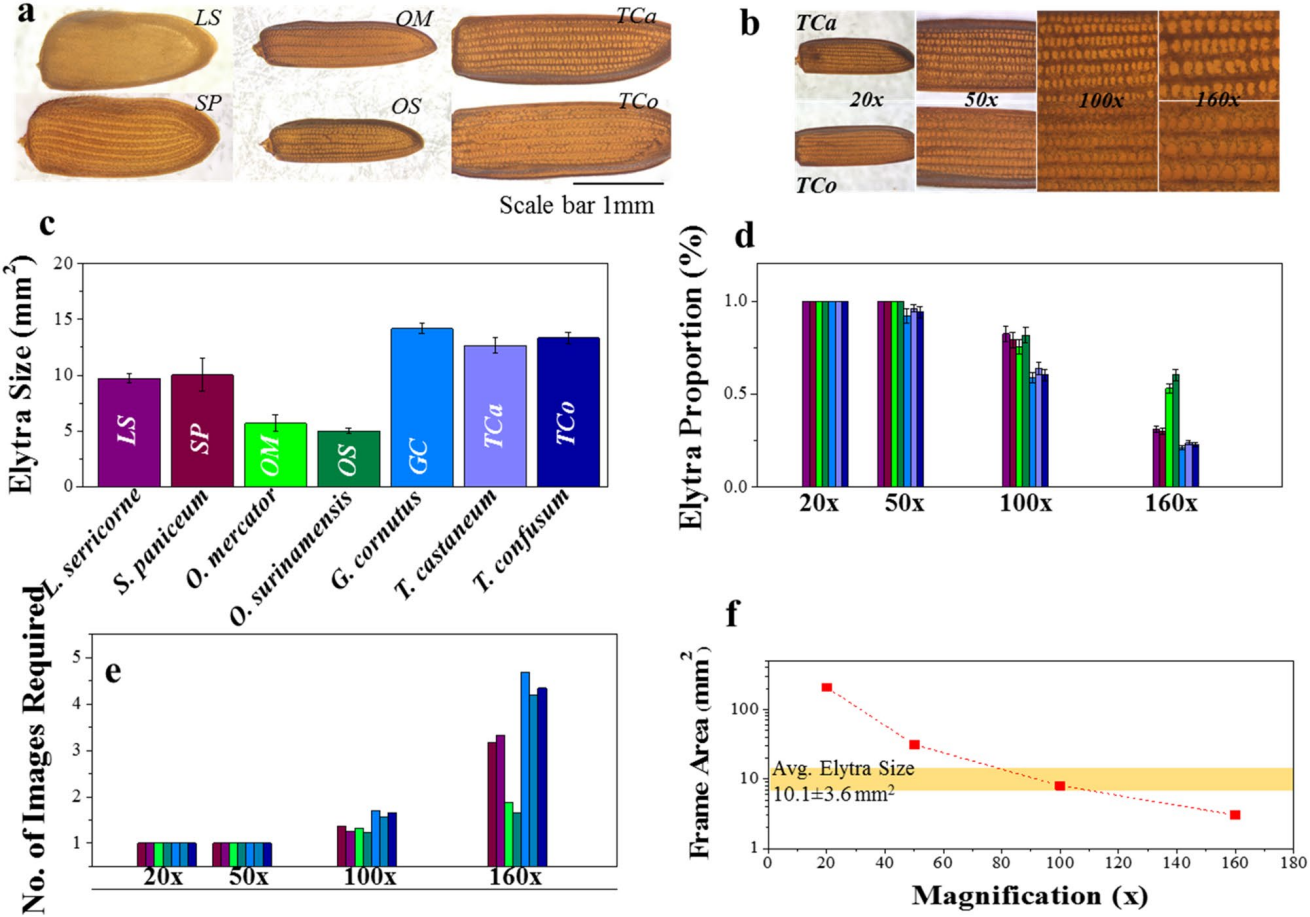
Magnification considerations: **(a)** Elytra images captured at 50 ×  magnification, showing their overall appearance; **(b)** Elytra images of *T. castaneum* (*TCa*) and *T. confusum* (*TCo*) at various magnifications showing area of elytra captured in each frame/magnification, **(c)** variation in average elytra sizes (of 10 images per species) for the seven different species used in this study, **(d)** proportion (area imaged/total area) of elytra captured at various magnifications, **(e)** number of images required (elytra size/frame size) to visually represent the whole elytra and **(f)** change in frame area (area captured in an image) by magnification. Even though there is a variation in elytral sizes, collectively they can be represented effectively at a magnification of 100 x.

Having studied the illumination systems, we focused on another important imaging parameter, namely, magnification. Figure 4b shows images of elytra from *O. mercator* and *O. surinamensis* at magnifications 20 x to 160 x under the *Trans* system. It is obvious that the patterns are visibly better and more prominent at higher magnifications. Moreover, the size and shape of an elytron varies from one species to another (Fig. 4a,c). For the seven beetle species examined in this study, their sizes varied from ~ 5 mm^2^ for *genus Oryzaephilus* to ~ 13 mm^2^ for *genus Tribolium*. At these sizes, 50 x to 100 x magnification seemed a good choice, as it enabled us to capture most of the elytra (Fig. 4b,d). Imaging at higher magnifications (such as 160 x) allowed significantly better visualization of individual patterns. On the other hand, it only revealed parts of the elytral patterns, with a large portion remaining out of the *field of view,* and thus unanalyzed.

**Figure 5:**
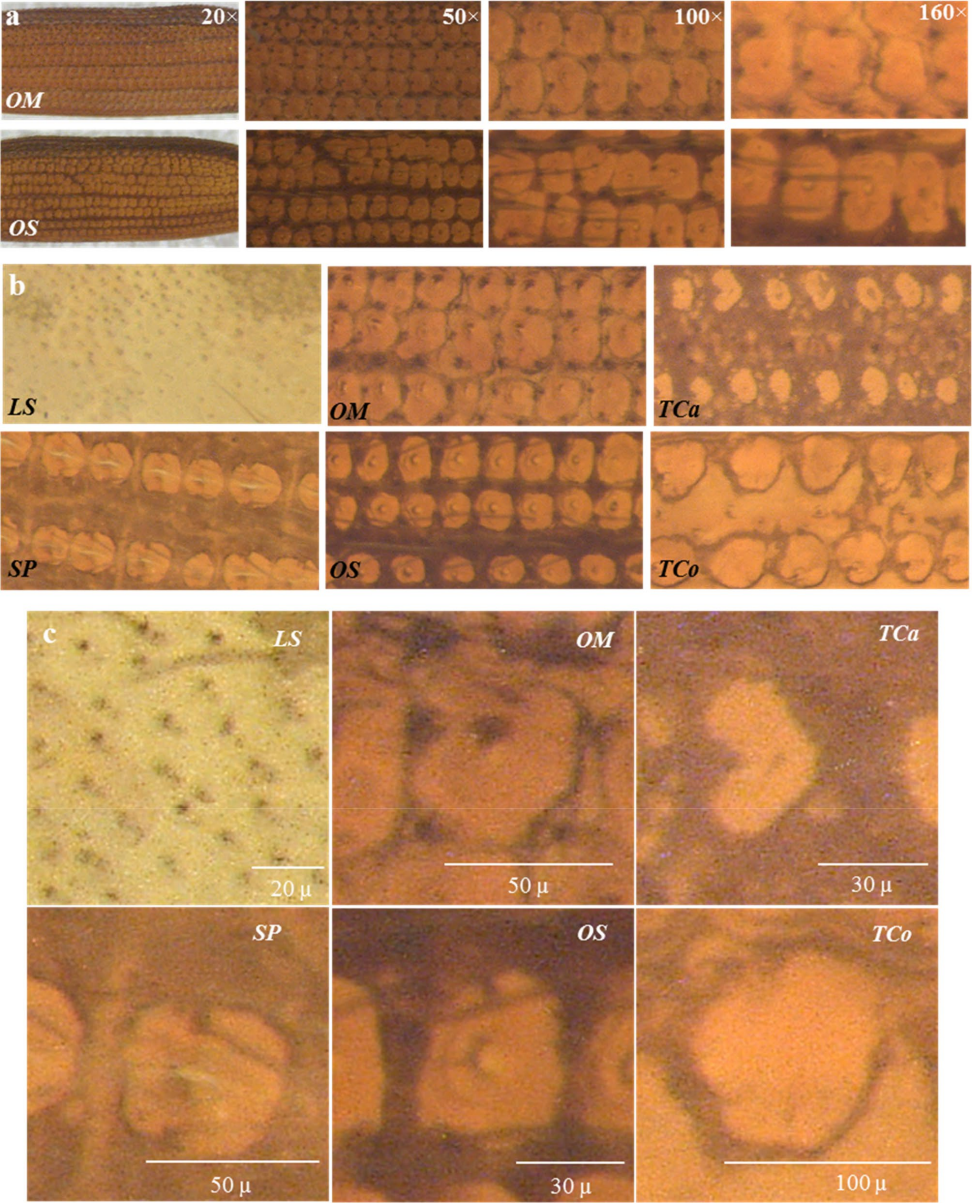
Elytra and their patterns at various magnifications; **(a)** elytral images of species *O. mercator (OM)* and *O. surinamensis (OS)* at magnifications 20x to 160x; **(b)** elytra patterns captured for six different species at 100x magnification under the *Trans* illumination system; and **(c)** various elytra patterns for the same six species at higher display resolution, which highlights that this setup (*Trans* at 100x) is capable of resolving the finest pattern details required to distinguish species belonging to the same genus or family from one another.

To remedy this inconvenience, many images must be captured in order to visually represent the entire elytra (about five or so in some cases as shown in Fig. 4e). This would increase overlapping errors (*i.e.* same parts being imaged in multiple images) without significantly increasing the pattern information. It also makes the process more susceptible to vibrations, thus increasing the likelihood of introducing undesirable artifacts. Increasing the magnification also significantly reduces the frame area. The average size of pantry beetle elytra is approximately 10 mm^2^, which is similar in dimension to the area of the image frame at 100 x magnification (Fig. 4f). Therefore, images captured at this magnification (i.e. 100 x), would reveal all or most of the elytra structure for a majority of the pantry beetles (Fig. 5a). We thus concluded that this a good starting point for a more detailed investigation.

Like the sizes of elytra, morphology of the patterns also varied widely across *family* and *genera*, and even within the same *genera,* which could clearly be visible at 100 x magnification across all the species (Fig. 5b). For instance, the central bulges were more circular in shape and measured on average ~ 100 µm diameter for *T. confusum* compared to a less circular bulge shape that measured less than ~ 50 µm in diameter for *T. castaneum*. Interestingly, *L. serricorne* completely lacked any such bulges (a feature we observed on very few pantry beetles) and had distances of less than 20 µm between the *seatal* pits (hair roots). Such small feature size could not be resolved adequately at 50 x magnification (even at ~ 2400 × 1900 dpi image resolution). Imaging at 100 x magnification (and at ~ 2400 × 1900 dpi resolution) allowed us to visualize the patterns clearly enough to visibly distinguish one elytra pattern from another (Fig. 5c). We therefore, chose this magnification as optimal, as it struck a reasonable balance between appropriate field of view and adequate resolution of detailed elytral patterns. At this point we also began to concur that magnification of 100 x under *Trans* illumination yielded high quality elytra images that allowed one to visualize fine patterns in beetle elytra in a consistent manner. It could be the optimal imaging condition, as images captured this way enabled us to visually differentiate one species from another, at least for the set of beetle species under investigation.

After optimizing the illumination and magnification settings (which were the hardware-based parameters), we focused our attention to digital parameters for improving the image quality. Amongst these parameters, sharpness, distortion and aberration were important for their significance in image processing. They also contribute to the visual clarity, by revealing the finest elytral patterns required for distinguishing one species from another. The corresponding FFT images of the images obtained using conventional (low resolution *2Pt_Rf*), *2Pt-Rf* and *Trans* settings (Supplementary Figure S5) highlights the difference in the pattern clarity. The central vertical line represents the horizonal groves in the pattern and the smaller spots on both sides possibly represent the rectangular box like features patterns. Their distinct nature for the FFT image, indicates that the pattern (obtained using the *Trans* setting) is clear due to the good image quality. On the contrary, the FFT patterns are increasingly diffused (due to glare and scattering, less sharpness and clarity) for *2Pt-Rf* setting and lower resolution. The FFT patterns from species belong to different families shows variation. The FFTs from those belong to the same genus and family, shows some similarity (due to the similarity in their elytral patterns) but are not identical, further bolsters the superior image quality of *Trans* setting that allows distinction of one species from another.

Image resolution, another of the image quality parameter, was kept at 2592 × 1944 dpi (~ 5-megapixel, image size: 14 MB per image). This was found to be adequate for our applications, as higher resolution (greater than 10 megapixel) increased the images size significantly without providing any additional information. This may also cause difficulty in handling and storage for a repository aimed to contain about 2000 images. Fortunately, most modern cameras, including the one we used, comes with well-documented, user-guided software interface that allows convenient optimization of the ‘soft’ parameters enabling easy capturing of good quality images. So, it was less arduous for us to obtain good quality elytral images of once the illumination and magnification settings were worked out. A step-wise details on how to capture a good quality image has been elaborated (Supplementary Information-Step-Wise Imaging Method) for consistency and reproducibility.

**Figure 6:**
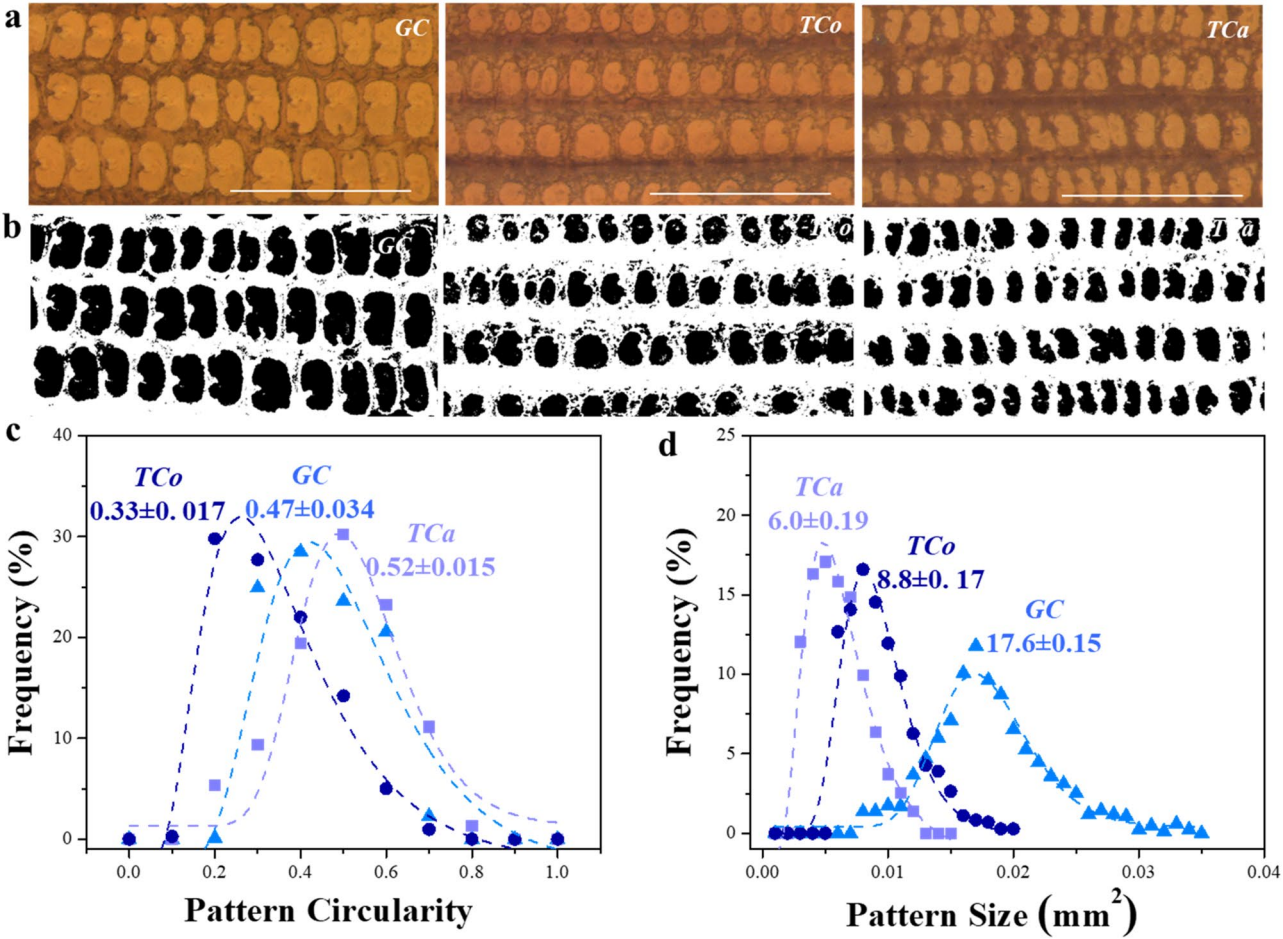
Analysis of elytra patterns for 3 different species, namely *G. cornutus* (GC), *T. confusum (TCo), & T. castaneum* (*TCa*) belonging to the same *family Tenebrionidae.* **(a)** optical images captured in *Trans* illumination at 100x magnification showing elytra patterns, with the scale bars being 250 µm; **(b)** the corresponding binary image, that shows only the dominant patterns. The log-normal distribution of **(c)** ‘circularity’ (shape) and **(d)** ‘size’ of the patterns for the three different species. It can be noted that the shape of the patterns are similar (close ‘circularity’ distribution) for the species belonging to the same *family*. But the ‘size’ distribution varied for three species with *TCo & TCa* belonging to the same *genus Tribolium*, much closer compared to the other family member *GC.* The indexed numbers show the mean and SD of the distribution, with the size values bearing units of  10^–3^ mm^2^.

Our ultimate objective is to obtain large number of high-quality elytral images for species identification through elytral pattern recognition using artificial intelligence (AI) based machine learning methods. However, developing such methods require both time and high-performing computational capability, which could be expensive. It may be prudent to run a ‘quick-check’ to observe if improving the image quality indeed showed any promise in improving species-level identification. Therefore, as a proof of concept, we used ImageJ to analyze the elytral patterns based on their difference in sizes and shapes. For this test, we analyzed elytra images of *G. cornutus, T. castaneum* and *T. confusum*, all three belonging to the same family *Tenebrionidae*, thus showing very similar elytral patterns (Fig. 6a). The processed images yield corresponding pattern outlines whose size and shape have been quantified by the area within the outlines and their circularity (area/perimeter^2^, 0 for line and 1 for circle) (Fig. 6b). It can be noted that their distributions created three different bell curves for the three species analyzed. The shape distributions did show three peaks having good overlap, probably indicating the similarity in pattern shapes for species belonging to the same family (Fig. 6c). The size distribution curves however are further apart for the species belonging to the same *family* but different *genus* and quite close for those of the same *genus* (Fig. 6d). This species wise classification using rudimentary size analysis of elytra patterns indicated that improving image quality may indeed help improve species-level identification.

**Figure 7:**
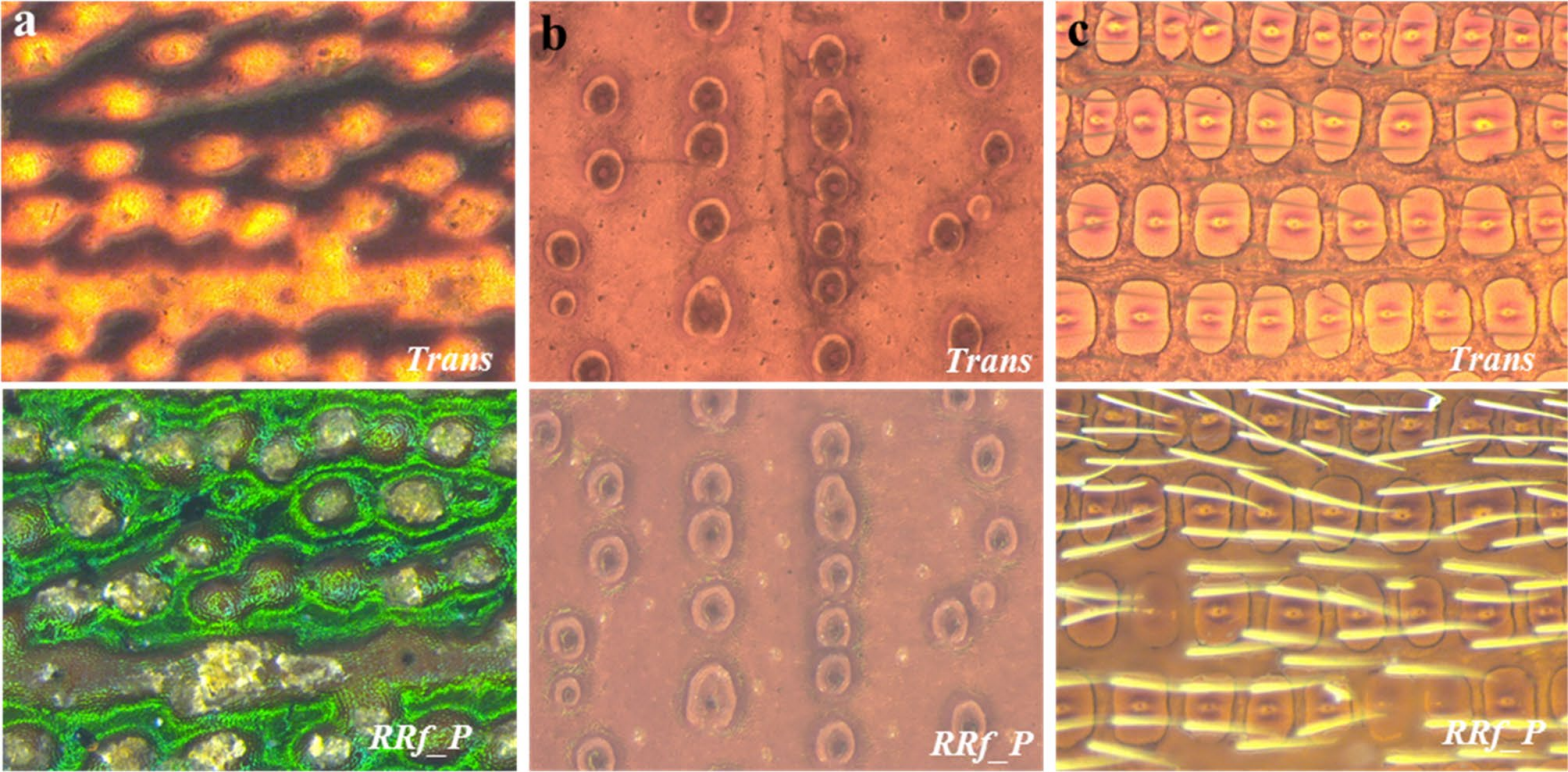
Control study to evaluate imaging capability when using *Trans* at 100x magnification, which was employed to image **(a)** Rainbow Scrap Beetle (*Phanaeus vindex*); **(b)** Black Dung Beetle (*Onitis aygulus*) and **(c)** Click Beetle (*Orthostethus infuscatus*), which possess some of the thickest and darkest known elytra in beetles. For comparison, the bottom row imaged using the *RRf_P* system, shows colored pigmentation, waxy coating and excessive setae. The top row represents images captured using the *Trans* system showing only the cytoskeletal structure, and which remain mostly unaffected by nonuniform surface features.

Any optimization technique must be tested for its robustness before being extended to a much wider range of samples. Thus, we wanted to observe whether the *Trans* setting at a 100x magnification could be used for other species of beetles. For this setting to work the transmitted light must pass through the elytra, which may seem difficult if the elytron is too dark and/or thick. Thus, we tested this system to image elytral patterns on rainbow scrap beetle (*Phanaeus vindex*), black dung beetle (*Onitis aygulus*) and click beetle (*Orthostethus infuscatus*), (none of which are pantry beetles), for they have some of the biggest, darkest and thickest elytral known to entomologists (Fig. 7a,b)^36^. It was indeed possible to capture elytra patterns for all three species using this setup. For comparison, images also were obtained using the *RRf_P* setting further highlighting the advantage of imaging in transmitted light, *Trans* remains least influenced by the presence of surface features such as pigmentation, excessive setae and the like as shown in the bottom row images in Fig. 7a–c. Since the setup worked well for some of the biggest and darkest elytra samples, presumably it could be used for imaging any species of pantry beetle (or other class of beetles) which are roughly 10 times smaller in size and have far thinner elytra.

**Figure 8:**
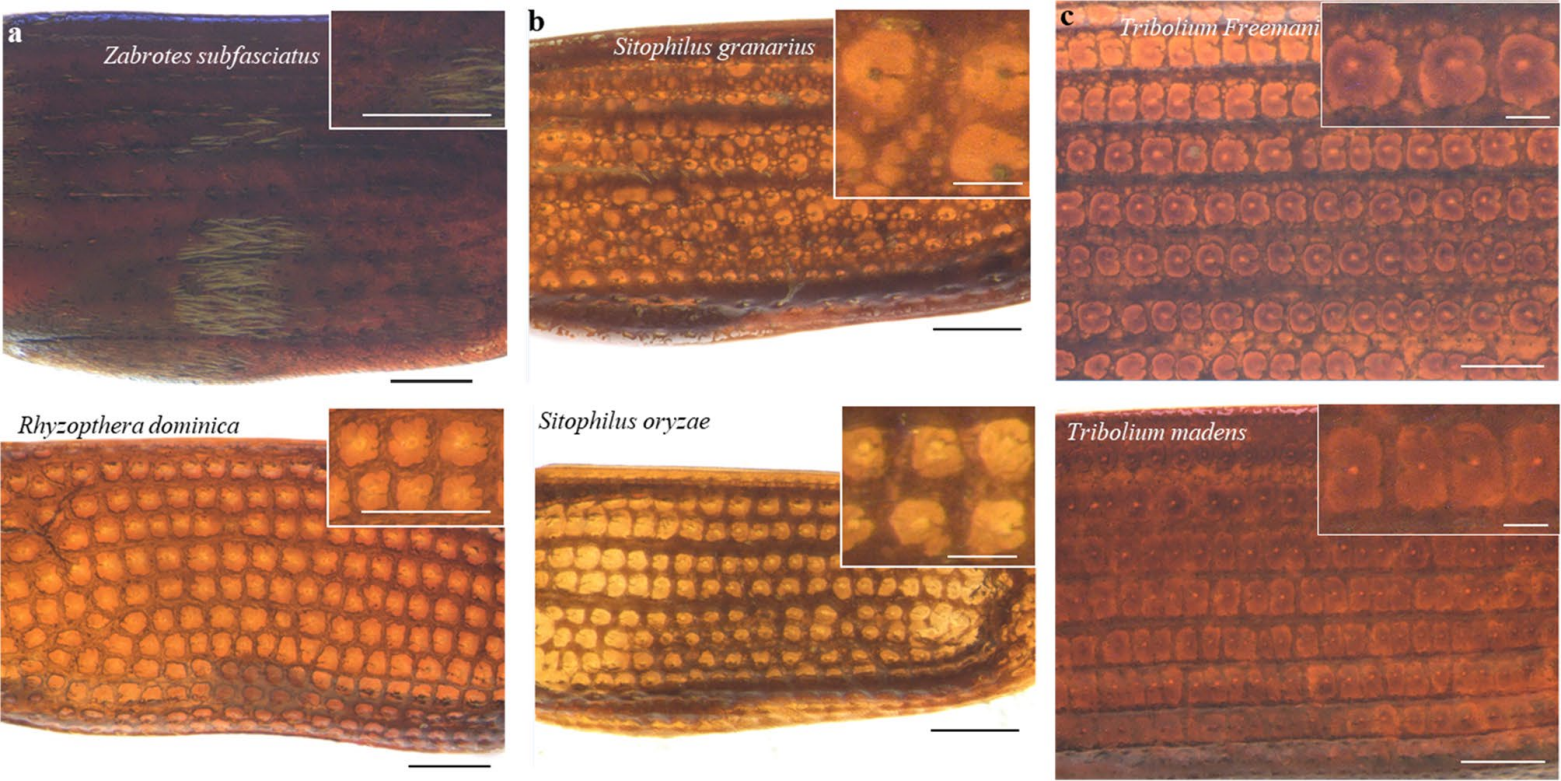
Extending this imaging method to other species of food contaminating beetles. Elytra images of species belonging to **(a)** two completely different families; **(b,c)** of same genus. *Sitophilus granarius* and *Sitophilus oryzae* of *genus Sitophilus*; *T. freemani* and *T. madens* of *genus Tribolium*. The optimized imaging technique works well for imaging elytra of most species of pantry beetles. However, species with variegated dorsal setae and deep concave shapes are most difficult to image properly. The images are at 100x magnification, with the scale bars being 250 µm; the insets show the magnified patters with scale bar being 100 µm.

Having tested the *Trans* system at 100x magnification on thicker and darker elytral samples, we moved forward in capturing elytral images of other pantry beetles to further expand the robustness of this system. Figure 8 shows representative images of six more species, listed in Table 1, by their *family* and *genus* names. We observed that this system (100 x magnification with the *Trans* setting) yielded excellent images for most of the beetles. The only exception was *Zabrotes subfasciatus* (*Z. subfasciatus-* commonly known as Mexican bean weevil of family *Chrysomelidae*). Its dark *dorsal setae* with white stripes, combined with a deeply concave shape probably made auto-focusing difficult during image acquisition. This could have contributed to slightly imperfect stacking of multi-layer images resulting in a poorer 3D montage construction. Images of the same species obtained through the *RRf_P* system also were of poorer quality, leading us to conclude that elytra with surface *setae* of contrasting colors combined with deep concave shapes are more difficult to image compared to elytra with more uniform color, flatter shape and clear patterns. This, however, may not pose a significant challenge, as in a real-world scenario, elytra often lose both their dorsal *setae* and concave shape due to food processing steps and/or fragmentation.

We currently are extending this imaging method to a total of 40 different species and plan to create a publicly available database of these images. Such database is meant to serve the food safety and entomology communities by providing good quality images for referencing and taxonomical applications. The optimization of parameters required for machine learning methods for species identification through pattern recognition are quite different from those in image acquisition and is far beyond the scope of this manuscript. But this study is aimed to serve as a forerunner for such study as it provides the means to obtain a large set of good-quality, noise-free images for developing a good computational model, which has been one of the primary challenges in the field of AI and machine leaning. We hope that a publicly available database of images will encourage data scientists to develop state-of-the art species identification algorithms, thus eventually contribute to our long-term goal of efficient automated species identification of food contaminating beetles. We believe this work lays a solid foundation for such an exhaustive study, which would require more resources and would help predict the correct species of pantry pests with greater accuracy. We hope such efforts ultimately would expedite the entire process of taxonomical analysis and help better manage contamination scenarios or catalog ecological systems, in the foreseeable future.

## Conclusion

Our study’s aim was to develop better imaging conditions that would allow capture of high-quality images of beetle elytra in a consistent manner. In this investigation, multiple species that had significant similarities (within a pair) were imaged under different illumination systems (i.e. *2Pt_Rf*, *RRf_P* and *Trans*) and various magnifications (i.e. 20 x to 160 x). Through this exhaustive imaging exercise, we sought to find the optimal imaging method that yielded images clear enough to differentiate the species belonging to the same *family* or *genus*. Of the three lighting conditions, we found the *Trans* setting was the most simple and economical, and also provided images that were least susceptible to external and random variables. We also found that both 50 x and 100 x magnifications were adequate to capture elytra specimens for most species of pantry beetles. However, 100 x magnification was more advantageous in resolving finer patterns whose dimensions sometimes were smaller than ~ 20 µm. Images captured using the optimal imaging condition, (i.e. the *Trans* setting at 100 x magnification) showed very clear elytral patterns. These images were also subjected to quick and simple pattern analysis to obtain their *size* and *circularity* distributions, which showed distinct distribution peaks specific to each species belonging to the same *family* or *genus*. It thus allowed us to capture high-quality images from various food-contaminating species and managed to highlight the subtle yet distinct differences in their elytra patterns, even for species that appeared nearly identical. We believe that the study has paved the way forward towards an automated species identification of food-contaminating (or of other types) beetles through elytral pattern recognition using advanced machine learning algorithms in the near future.

## Supplementary Information

Supplementary Figures.
