# Supplementary material for: Optimized imaging methods for species-level identification of food-contaminating beetles
Source: Sci Rep. 2021 Apr 12;11:7957. doi: 10.1038/s41598-021-86643-y (PMC8041796; doi:10.1038/s41598-021-86643-y)
Supplement: Supplementary file 1 — Supplementary Figures. [file 41598_2021_86643_MOESM1_ESM.docx]

**Optimized Imaging Methods for Species-Level Identification of Food-Contaminating Beetles**

Tanmay Bera^1^, Leihong Wu^1^, Hongjian Ding^2^, Howard Semey^2^, Amy Barnes^2^, Zhichao Liu^1^, Himansu Vyas^2^, Weida Tong^1^, Joshua Xu^1*^

1. Division of Bioinformatics and Biostatistics, National Center for Toxicological Research (NCTR), Food and Drug Administration (FDA), Jefferson, AR 72079, USA

2. Food Chemistry Laboratory-1, Arkansas Laboratory (ARKL), Office of Regulatory Sciences, Office of Regulatory Affairs (ORS/ORA), FDA, AR 72079, USA

* Address correspondence to: Joshua Xu, (email: Joshua.Xu@fda.hhs.gov)

**Disclaimer:**

The views expressed in this work are those of the authors only and do not necessarily express the views/policies of the U.S. FDA. The mention of trade names or specific manufacturers’ products are for clarification and should not be considered endorsements.

**Supplementary Information:**

**Supplementary Figure S1:**

**
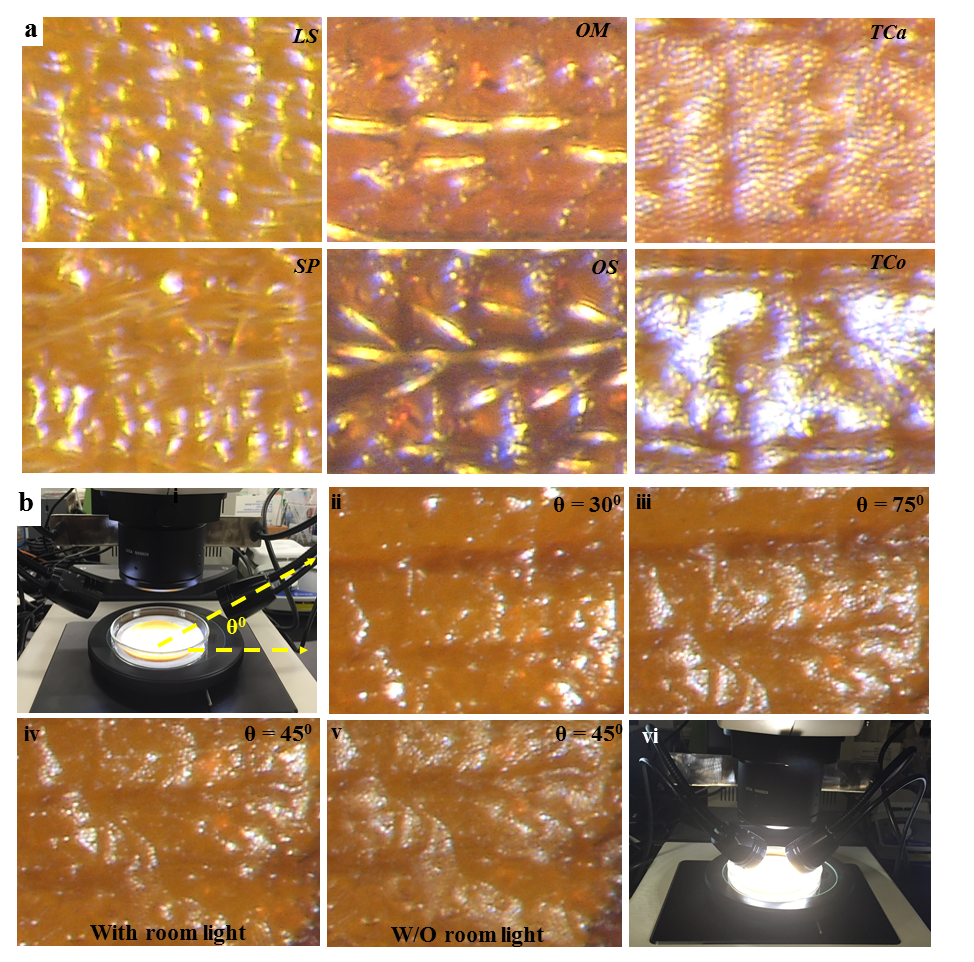
**

**Supplementary Figure S1:** Disadvantages of the ***2Pt_Rf*** system a: elytra from six different beetle species showing glaring reflections. Images were captured at 100× magnification; b: dependence on angle of incident light (*i* to *iv*) and ambient light (*iv* to *vi*), as observed in imaging of ventral-side of *T. catenium (TCa)*, elytra, where glare was minimal.

**Supplementary Figure S2:**


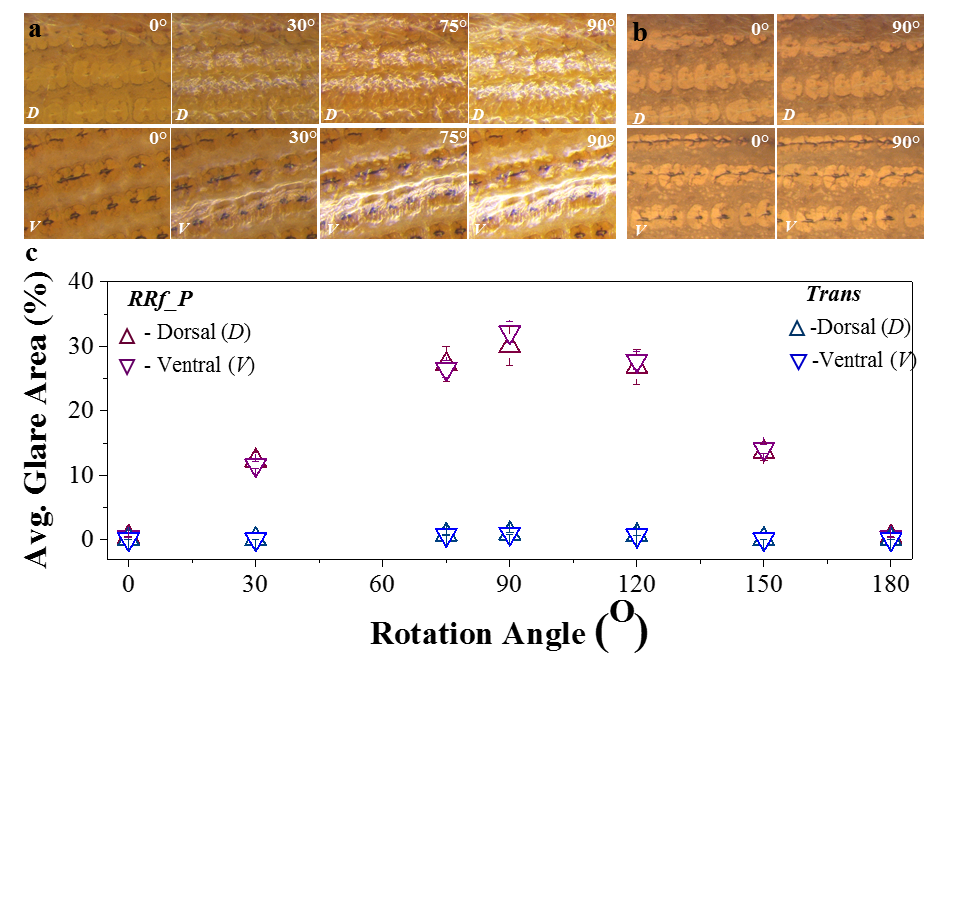


**Supplementary Figure S2:** Effect of optical filter: images of *S. paniceum* elytra (both dorsal and ventral sides) captured under a: ***RRf_P*** and b: ***Trans*** light, with the optical filter at various orientation angles; c: the variation in Average Glare Area (%) with the change in angular orientation of the optical filter for both illumination systems. Note that the ***RRf_P*** is more dependent on external factors, such as the angle of the optical filter, which make it a less consistent option.

**Supplementary Figure S3:**


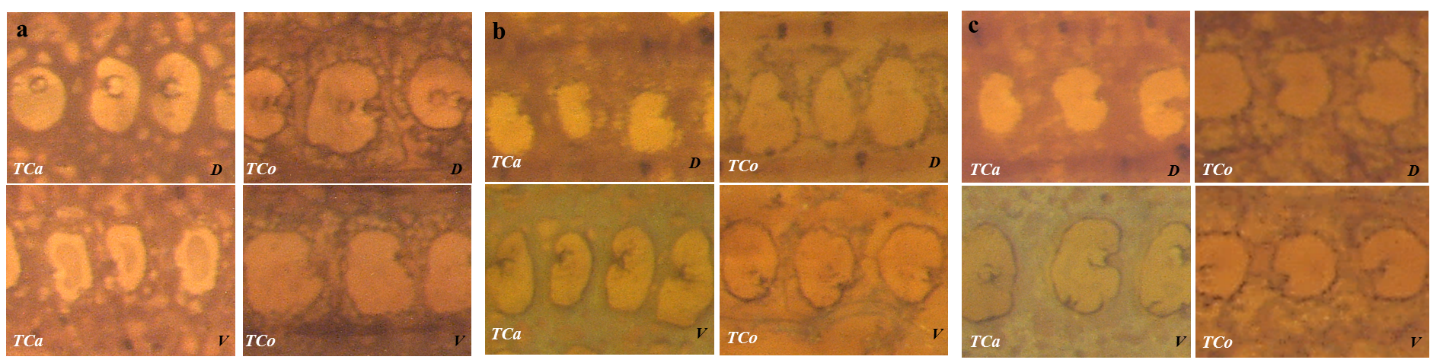


**Supplementary Figure S3:** Exploring combined illumination of the ***RRf_P*** and ***Trans*** systems. Elytra images of *T. castenium* (*TCa*) and *T. confusum* (*TCo*) at 100× magnification captured under a: ***Trans***, b: ***Trans + RRf_P*** and c: ***RRf_P***. Attempting to combine both ***Trans*** and ***RRf_P*** systems to create a fourth lighting system, namely ***Trans + RRf_P***, did not help in capturing both surface and internal features with equal clarity.

**Supplementary Figure S4:**


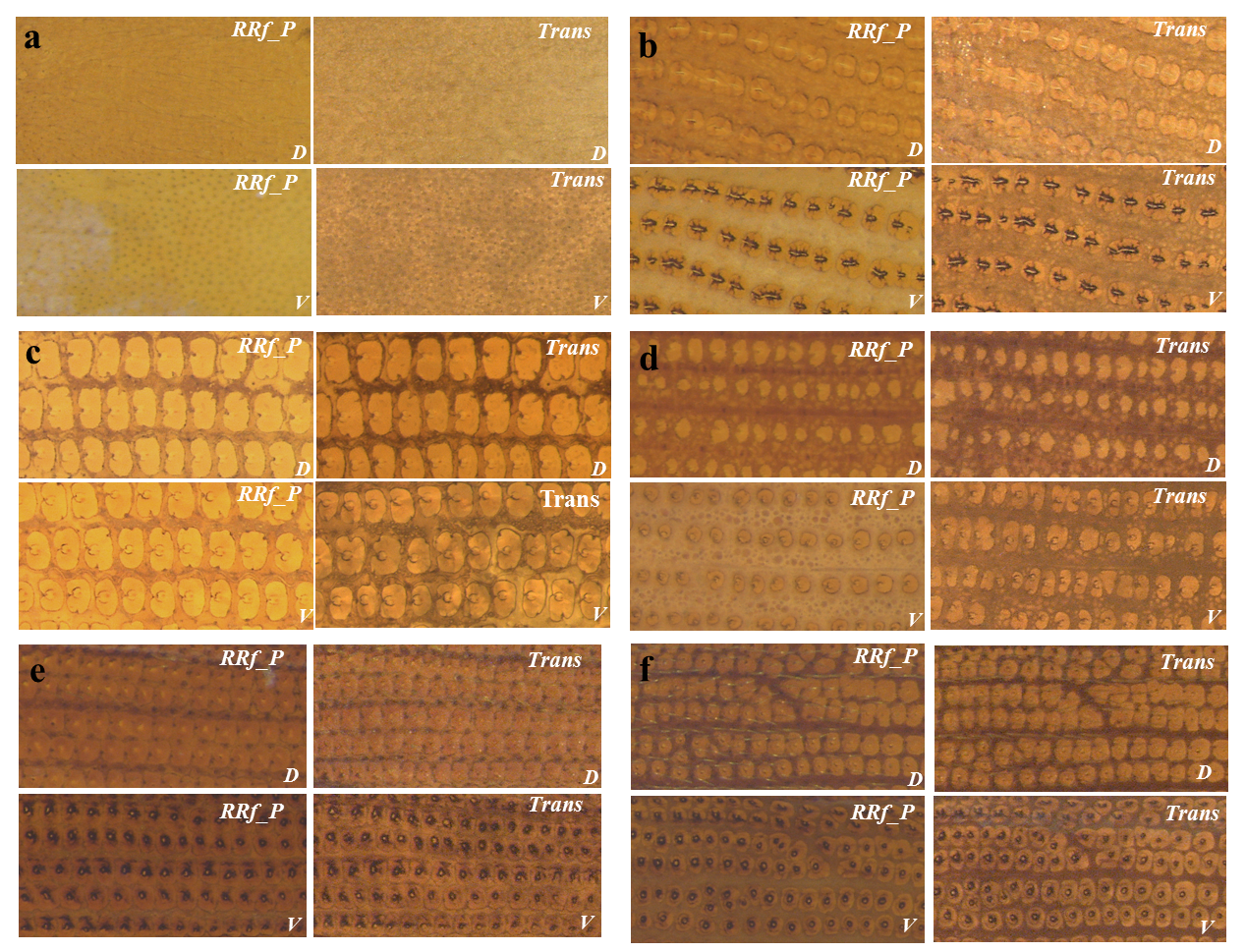


**Supplementary Figure S4:** Imaging of both *dorsal* (*F*) and *ventral* (*B*) sides with illumination systems ***RRf_P*** and ***Trans*** to observe maximum features in elytra images, for six different species: a: *L. serricorne* (*LS*)*, b: S. paniceum* (*SP*), c: *G. Cornutus* (*GC*), d: *T. castaneum* (*TCa*), e: *O. mercator* and f: *O. surinamensis.* It can be noted that species *LS* & *SP,* belonging to the *family* *Anobiidae*, when imaged without any glare, can easily be distinguished due to the variations in their patterns and surface structures. The same is also true for *GC* and *TCa*, belonging to the *family* *Tenebrionidae*, suggesting that family-level differentiation is relatively easy when either the ***RRf_P*** or ***Trans*** systems are used. It is slightly more challenging to distinguish between those species which belong to the same genus, as can be seen for *OM* and *OS* of *genus* *Oryzaephilus.*

**Supplementary Figure S5:**

**
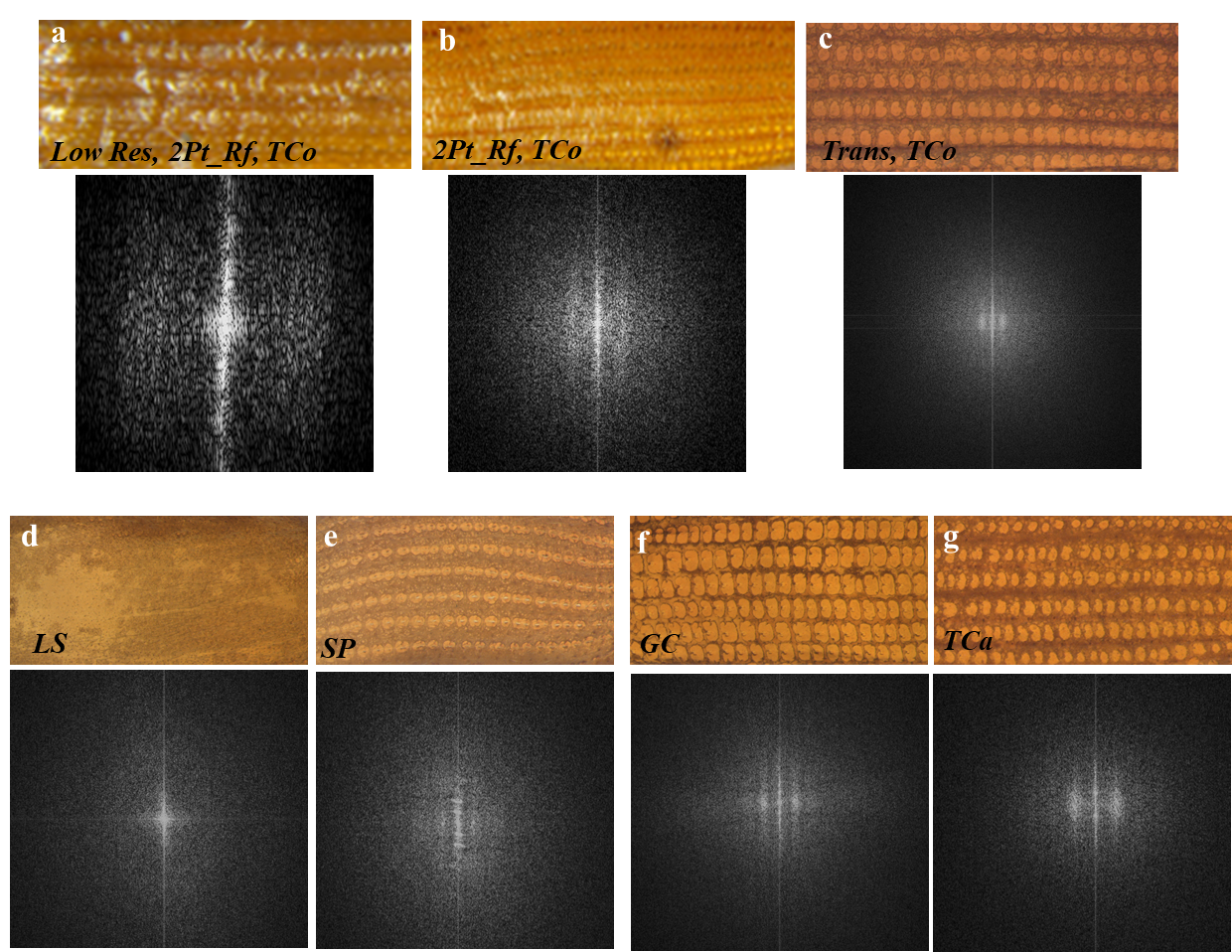
**

**Supplementary Figure S5:** Difference in elytral image quality through FFT analysis. Top row elytral images and below them are their corresponding FFT images for a: conventional low resolution (using 2Pt_Rf), b: 2Pt_Rf and c: Trans. The distinct and clear FFT pattern highlights the high image quality acquired through Trans setting.

The bottom panel shows the elytral images and their corresponding FFT (central part) for d: *L. serricorne* (*LS*)*, e: S. paniceum* (*SP*), f: *G. Cornutus* (*GC*), g: *T. castaneum* (*TCa*), showing the distinct FFT patterns corresponding to every species (due to the different elytral patterns obtained through ***Trans*** setting) even for species belong to same genus and/or family.
